# Risk factors for cancer among patients with type 2 diabetes: a retrospective cohort study

**DOI:** 10.1186/s12885-025-14483-4

**Published:** 2025-07-01

**Authors:** Hui Liu, Tian-Yi Zhang, Ya-Wen Wang, Juan-Juan Gao

**Affiliations:** 1 Biobank, The First Affiliated Hospital of Xi ’an Jiaotong University, Xi’an, Shaanxi 710061 China; 2 Shaanxi Engineering Research Center for Biobank and Advanced Medical Research, The First Affiliated Hospital of Xi’an Jiaotong University, 277 West Yanta Road, Xi’an, Shaanxi 710061 China

**Keywords:** Neoplasms, Diabetes mellitus, type 2, Risk factors, Biomarkers, Retrospective cohort study

## Abstract

**Background:**

Type 2 diabetes mellitus (T2DM) and cancer pose significant public health challenges worldwide. This study investigated the distribution of cancer cases and associated risk factors among patients with T2DM, aiming to inform clinical practices and reduce the burden of co-morbidities.

**Methods:**

A retrospective cohort study analyzed electronic medical records of 70,073 T2DM patients admitted between 2013 and 2019. After exclusions, 3,284 individuals were included, with a median follow-up of 27 months. Associations between 38 baseline characteristics and cancer risk were first assessed using univariate Cox proportional hazards models (reported as hazard ratios [HRs] with 95% confidence intervals [CIs]). Significant predictors (*p* < 0.05) were further analyzed in adjusted multivariable Cox models by backward elimination (retaining 2 significant covariates).

**Results:**

Among the participants, 467 (14.2%) developed cancer, with digestive system cancers being the most prevalent (5.8%). The median age of participants was 58 years, with 59% being male. Risk factors for cancer among T2DM patients identified in multivariate analysis included lower serum uric acid levels (HR: 0.9970, 95% CI: 0.9942–0.9998), and higher AST/ALT ratio (HR: 1.84, 95% CI: 1.16–2.92). Specific risk factors for different types of cancer were also identified, such as lower apolipoprotein A for digestive system cancers and older age and lower serum uric acid levels for lung cancer.

**Conclusions:**

These findings highlight the potential value of integrating cancer screening into T2DM management for high-risk patients, such as individuals with altered uric acid or liver enzyme profiles. However, the study’s retrospective design and reliance on single-center data necessitate further prospective studies to validate these associations and refine biomarker-guided monitoring strategies.

**Supplementary Information:**

The online version contains supplementary material available at 10.1186/s12885-025-14483-4.

## Background

Type 2 diabetes mellitus (T2DM) and cancer are major global public-health concerns. In the next two decades, the global burden of both diseases will rise by approximately 60% [1]. In 2022 alone, nearly 20 million new cases and 9.7 million deaths [2] were reported, while in 2021, around 537 million adults had diabetes, causing 6.7 million deaths [3]. By 2045, the number of adults with diabetes is expected to reach 783 million, mainly in low- and middle- income countries [3]. Notably, while cancer incidence has declined in non-diabetic populations, it has remained stable or even increased in T2DM patients [4], underscoring the need to investigate their interconnection.

Epidemiological studies and meta-analyses have linked T2DM to elevated risks of pancreatic, colorectal, live, gallbladder, prostate, bladder, and cervical cancers [5–13]. Notably, the risk of pancreatic cancer is increased approximately 2- to 8-fold [12]. Similarly, T2DM is associated with a 23% higher risk of colorectal cancer, and a 58% elevated risk of liver cancer [13]. For genitourinary cancers, T2DM confers a 21% increased risk for bladder cancer and a 38% higher risk for cervical cancer [13], while the association with prostate cancer appears to be negative [9]. These studies underscore diabetes as a significant risk modifier for multiple cancer types.

Shared pathophysiological mechanisms, such as insulin resistance, chronic inflammation, oxidative stress, and obesity, underlie their connections [14, 15]. Specifically, chronic hyperglycemia fuels a pro-inflammatory state characterized by elevated cytokines (e.g., TNF-α, IL-6), which activate signaling pathways like NF-κB and JAK/STAT, promoting tumor cell proliferation, survival, and angiogenesis [16]. Hyperinsulinemia, a hallmark of T2DM, directly exerts mitogenic effects and enhances IGF-1 bioactivity, activating PI3K/Akt and MAPK pathways to inhibit apoptosis and stimulate cell proliferation, thereby increasing cancer risk [17–19]. These metabolic alterations create a tumor-friendly microenvironment, highlighting the urgency of developing effective screening and preventative strategies.

Despite these insights, early cancer detection in T2DM patients remains challenging due to the heterogeneous clinical presentations of cancers. While prior studies have identified associations between individual biomarkers (e.g., serum uric acid, lipid profiles) and cancer risk in diabetic populations [20–22], few have developed a comprehensive model incorporating readily accessible, low-cost laboratory indices for cancer risk stratification in T2DM patients. Moreover, existing machine learning models for cancer prediction often lack clinical interpretability [5], limiting their translation to routine care.

To address these gaps, this retrospective cohort study aims to investigate cancer incidence patterns and identify key clinical biomarkers predictive of cancer risk among hospitalized T2DM patients. By comparing longitudinal outcomes between T2DM patients who developed incident cancers and those who remained cancer-free during follow-up, the study seeks to characterize actionable laboratory indicators that may facilitate early cancer risk stratification. By analyzing routinely available clinical biomarkers, we hope to provide insights for early cancer risk stratification, with the ultimate goal of informing targeted screening strategies and reducing the dual global burden of T2DM and cancer.

## Methods

### Participants

**Fig. 1:**
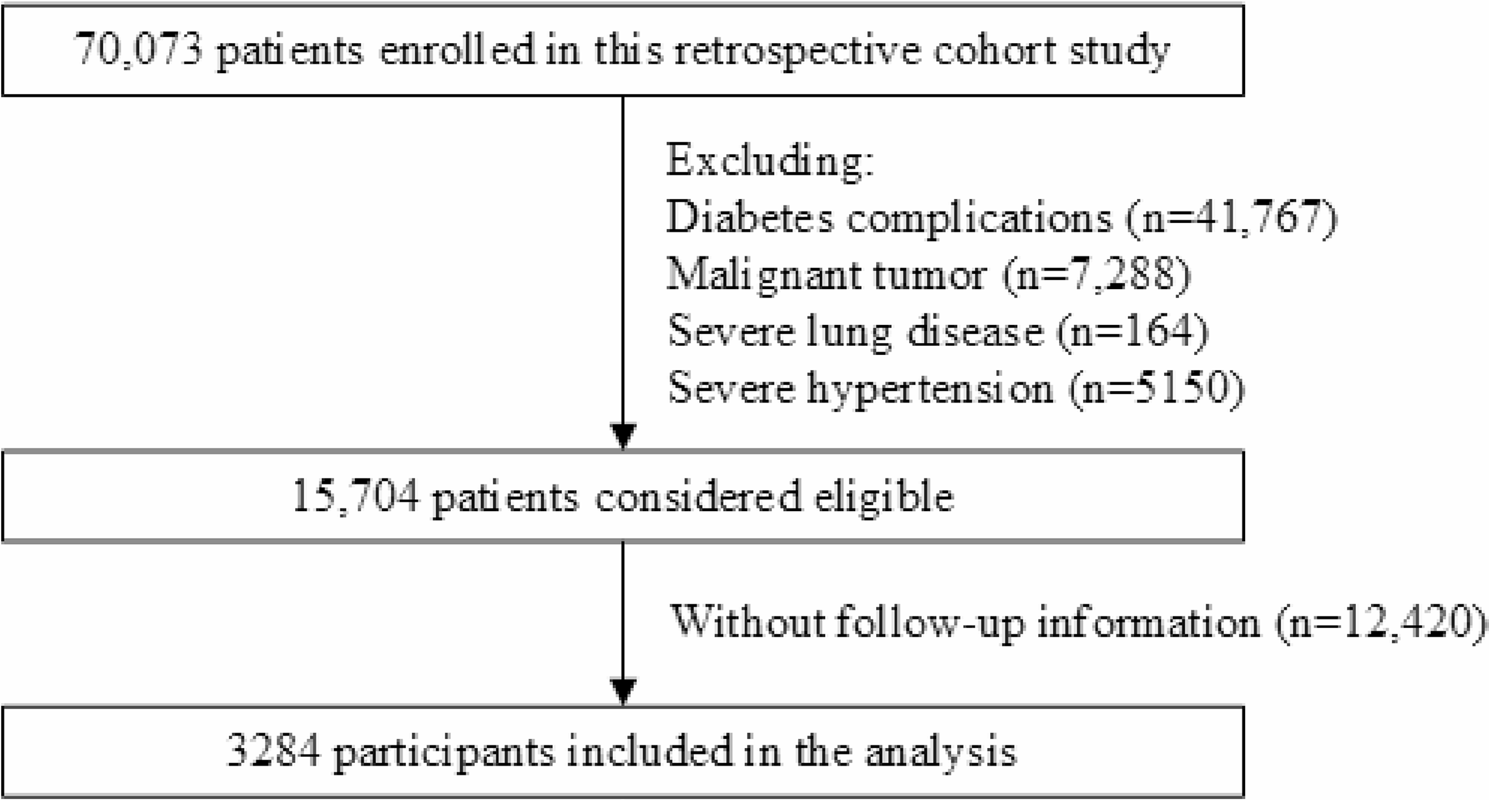
Flowchart of patient selection for this study

A retrospective cohort study was conducted among patients with T2DM who were admitted to the First Affiliated Hospital of Xi’an Jiaotong University between 2013 and 2019. The cohort entry date was designated as the index date, with patients identified to have pre-existing diabetes complications prior to this date being excluded. Consequently, a total of approximately 70,073 Chinese patients with type 2 diabetes were enrolled, from which 41,767 subjects were excluded due to diabetes complications (including diabetes retinopathy, heart failure, acute myocardial infarction, ischemic heart disease, coronary heart disease, atherosclerosis, cerebral infarction, peripheral neuropathy, cataract, chronic kidney disease, diabetic foot, traumatic amputation, foot ulcers, ketoacidosis, arterial embolism and thrombosis, and peripheral vascular disease). Additionally, 7288 patients with prior malignant tumors were removed. Since severe respiratory diseases and hypertension independently correlate with inflammation and metabolic dysregulation, which could obscure the direct relationship between T2DM and cancer risk, 5314 patients with these conditions were also excluded, leaving 15,704 eligible individuals for inclusion. After further exclusion of patients lacking follow-up data (*n* = 12,420), the final analytical cohort comprised 3284 patients (Fig. 1). This study was approved by the Institutional Review Board of the First Affiliated Hospital of Xi’an Jiaotong University (approval no. XJTU1AF2018LSL-009). All the clinical investigations were conducted according to the provisions of the Declaration of Helsinki.

### Data collection

All data were retrieved from the electronic medical records system, encompassing sociodemographic factors, diabetes-related variables, and biomarkers. These included age, sex, body mass index (BMI), smoking status, alcohol drinking status, duration of type 2 diabetes, systolic blood pressure, diastolic blood pressure, glycated haemoglobin A1c (HbAlc), fasting blood glucose, serum urea, serum creatinine, serum uric acid, cystatin C, estimated glomerular filtration rate (CKD-EPI), total cholesterol, triglycerides, low-density lipoprotein, high-density lipoprotein, lipoprotein(a), apolipoprotein A, apolipoprotein B, apolipoprotein E, albumin, total bilirubin, direct bilirubin, aspartate aminotransferase (AST), alanine transaminase (ALT), AST/ALT, total bile acid, glycated albumin, platelet count, lymphocyte, neutrophil, erythrocyte and mean corpuscular hemoglobin concentration. BMI was calculated by weight (kg)/height (m^2^), which were measured at admission. All patient identifiers were removed prior to analysis, and data were pseudonymized using unique study codes.

### Follow up

The follow-up for all patients was extended until December 2019, with a median follow-up period of 27 months (interquartile range: 6–56). The primary focus of these follow-ups included the disease status and occurrence of any type of cancer among all patients. Incident cancer cases were initially identified through ICD-10 codes from electronic health records, then confirmed histologically by reviewing pathology reports. Notably, all patients contributed comprehensive follow-up data, facilitating the accurate determination of outcomes from the time of discharge up until the final follow-up visit.

### Statistical analysis

**Fig. 2:**
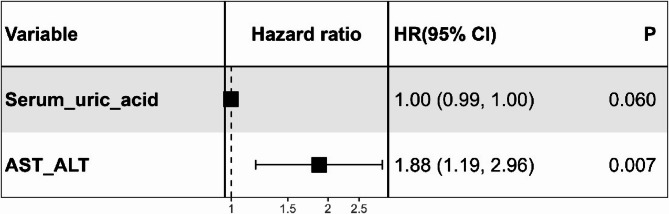
Forest plot of the multivariable Cox regressions analysis for cancer risk factors in patients with type 2 diabetes. Forest plot derived from the multivariable Cox proportional hazards regression analysis aimed at assessing cancer risk factors in type 2 diabetes. Each black square represents the point estimate of the hazard ratio (HR) for the corresponding variable, the horizontal lines extending from the squares indicate 95% CIs of the HRs, and the vertical dotted line at HR = 1 serves as a visual reference, representing the null effect. The HR and 95% CI for serum uric acid, rounded to four decimal places, was 0.9970 (95% CI: 0.9942–0.9998). Abbreviations: AST, aspartate aminotransferase; ALT, alanine transaminase; CI, confidence interval; HR, hazard ratio

We reported median with interquartile range (IQR) for continuous variables and proportions for categorical variables. Group differences in continuous variables were evaluated using the Wilcoxon rank sum test, and categorical data were compared using Pearson’s chi-squared test. For time-to-event analysis, the index admission date served as time zero, with censoring occurring at cancer diagnosis, the last hospital contact, or the study’s end (December 31, 2019). The proportional hazards assumption was validated through global and covariate-specific Schoenfeld residual tests [23], revealing no significant violations (all *p* > 0.05; Supplementary Table 1). Multicollinearity was assessed using variance inflation factors (VIFs). We employed univariable Cox proportional hazards regression models to explore the associations between 38 baseline characteristics and overall as well as type-specific cancer risks in T2DM patients. To account for multiple comparisons, a Bonferroni-corrected significance threshold of *P* < 0.0013 (α = 0.05/38) was applied. Hazard ratios (HRs) and their corresponding 95% confidence intervals (CIs) were calculated. Variables with *P* < 0.05 in univariable analyses were considered for inclusion in adjusted multivariable models, which were constructed using backward stepwise elimination based on the Akaike Information Criterion (AIC), and the results were visualized using Forest plots (e.g., Fig. 2). Final risk factors were selected based on sustained statistical significance (*P* < 0.05) in the multivariable analysis, adhering to established variable selection frameworks for survival analyses [24]. All statistical tests were two-tailed, and a *P* value of less than 0.05 was considered statistically significant. Data analyses were conducted using R software (version 3.6.1, Vienna, Austria).

## Results

### Participants

Among the 70,073 patients with T2DM, a total of 3284 eligible participants were included in the analysis. The median age of these participants was 58 years (IQR: 50–67), with 59% being male. After a median follow-up of 27 months (IQR: 6–56), 467 participants (14.2%) developed cancer. Patients with T2DM who developed any type of cancer were significantly older than those did not (*P* < 0.001, Table 1). Apart from serum uric acid, ALT, AST/ALT, and white blood cell counts, all other characteristics were comparable between the two groups (*P* > 0.05, Table 1).

**Table 1:** Baseline characteristics stratified by cancer occurrence

Variable	Overall^†^ *N* = 3,284	Patients with type 2 diabetes^†^		*P* ^‡^
		Non-cancer group *N* = 2,817	Cancer group *N* = 467	
Age (years)	58 (50, 67)	58 (49, 66)	61 (53, 68)	< 0.001
Sex				0.554
Female	1,337 (41%)	1,141 (41%)	196 (42%)	
Male	1,946 (59%)	1,675 (59%)	271 (58%)	
Smoking				0.801
No	2566 (94.2)	2213 (94.2)	353 (93.9)	
Yes	159 (5.8%)	136 (5.8%)	23 (6.1%)	
Alcohol drinking				0.966
No	2598 (95.6)	2240 (95.6)	358 (96.0)	
Yes	110 (4.1%)	95 (4.1%)	15 (4.0%)	
Body mass index (kg/m^2^)	24.2 (22.1, 26.4)	24.2 (22.1, 26.4)	23.9 (22.0, 26.3)	0.288
Systolic blood pressure (mm Hg)	127 (116, 140)	127 (116, 140)	129 (119, 141)	0.094
Diastolic blood pressure (mm Hg)	78 (70, 86)	78 (70, 86)	78 (71, 85)	0.389
HbA1c level (%)	7.20 (6.30, 8.70)	7.20 (6.30, 8.70)	7.10 (6.40, 8.70)	0.476
Fasting blood glucose (mmol/L)	7.4 (5.7, 10.7)	7.5 (5.8, 10.8)	7.1 (5.6, 9.7)	0.068
Low-density lipoprotein (mmol/L)	2.36 (1.80, 2.96)	2.35 (1.79, 2.96)	2.46 (1.86, 2.78)	0.921
High-density lipoprotein (mmol/L)	0.95 (0.78, 1.15)	0.95 (0.78, 1.14)	0.95 (0.81, 1.15)	0.767
Apolipoprotein A (g/L)	1.10 (0.95, 1.26)	1.11 (0.95, 1.25)	1.08 (0.94, 1.27)	0.479
Apolipoprotein B (g/L)	0.80 (0.65, 0.97)	0.81 (0.66, 0.97)	0.76 (0.64, 0.90)	0.162
Apolipoprotein E (mg/L)	36 (28, 49)	37 (29, 49)	34 (27, 45)	0.110
Lipoprotein(a) (mg/L)	102 (51, 202)	102 (52, 206)	100 (47, 164)	0.354
Duration of type 2 diabetes (years)	4.0 (0.9, 9.0)	4.0 (0.8, 9.0)	3.9 (1.0, 9.0)	0.996
Estimated glomerular filtration rate (CKD-EPI), mL/min	104 (94, 115)	104 (94, 115)	104 (94, 112)	0.692
Serum urea (mmol/L)	5.18 (4.15, 6.40)	5.18 (4.14, 6.41)	5.09 (4.19, 6.34)	0.869
Serum creatinine (µmol/L)	56 (46, 67)	56 (46, 67)	53 (45, 67)	0.131
Serum uric acid (µmol/L)	283 (226, 351)	286 (230, 354)	264 (209, 327)	< 0.001
Triglyceride (mmol/L)	1.49 (1.03, 2.24)	1.50 (1.04, 2.29)	1.37 (0.90, 1.98)	0.106
Total cholesterol (mmol/L)	3.94 (3.24, 4.70)	3.93 (3.23, 4.70)	4.00 (3.25, 4.67)	0.682
Platelet (10^9^)	180 (130, 230)	180 (132, 232)	174 (120, 223)	0.136
Albumin (g/L)	39 (35, 43)	39 (35, 43)	39 (36, 43)	0.831
Total protein (g/L)	66 (61, 71)	66 (61, 71)	66 (61, 71)	0.586
Cystatin C (mg/L)	0.87 (0.72, 1.07)	0.86 (0.72, 1.07)	0.87 (0.72, 1.07)	0.847
Total bilirubin (µmol/L)	13 (9, 20)	13 (9, 20)	14 (9, 18)	0.316
Direct bilirubin (µmol/L)	4 (3, 7)	4 (3, 7)	4 (3, 7)	0.107
AST (U/L)	21 (17, 33)	22 (17, 33)	21 (16, 31)	0.212
ALT (U/L)	24 (15, 39)	24 (16, 40)	22 (14, 35)	0.022
AST/ALT	0.91 (0.70, 1.27)	0.90 (0.70, 1.25)	1.00 (0.76, 1.30)	0.024
Total bile acid (µmol/L)	4 (3, 9)	4 (2, 9)	5 (3, 10)	0.104
Lymphocyte (10^9^)	1.50 (1.03, 2.03)	1.50 (1.03, 2.03)	1.46 (1.03, 1.95)	0.293
Neutrophil (10^9^)	3.86 (2.78, 5.50)	3.89 (2.80, 5.54)	3.68 (2.66, 5.13)	0.088
Mean corpuscular hemoglobin concentration (g/L)	334 (325, 342)	335 (326, 342)	333 (324, 343)	0.382
White blood cell (10^9^)	6.2 (4.7, 8.0)	6.2 (4.8, 8.1)	5.9 (4.7, 7.3)	0.027
Glycated albumin (%)	18 (16, 24)	18 (16, 24)	19 (16, 24)	0.290
Erythrocyte (10^12^)	4.41 (3.87, 4.81)	4.42 (3.87, 4.82)	4.36 (3.89, 4.78)	0.349

*Abbreviations:AST* Aspartate aminotransferase, *ALT* Alanine transaminase, *HbAlc* Haemoglobin A1c

^†^Median (IQR); n (%)

^‡^Wilcoxon rank sum test; Pearson’s Chi-squared test

### Risk factors for cancer among T2DM patients

Univariable Cox analysis identified several predictors of cancer risk in T2DM patients. After multiple test correction, older age was significantly associated with higher risk of cancer development (HR: 1.02, 95% CI: 1.01–1.03, *p* < 0.001). However, hepatic dysfunction marked by elevated AST/ALT ratio (HR: 1.20, 95% CI: 1.05–1.36, *p* = 0.007) and metabolic disturbances including lower triglycerides, higher glycated albumin, and reduced lymphocytes were associated with cancer risk (all *p* < 0.05; Table 2), requiring further validation. A sensitivity analysis was conducted by retaining 5,314 T2DM patients with severe respiratory diseases and hypertension. The results (Supplementary Table 2) demonstrated minimal differences compared to the original findings, with cancer risk variables that reached statistical significance remaining consistent with those in the primary analysis (Table 2).

**Table 2:** Univariate cox’s proportional hazards model analysis for cancer risk in patients with type 2 diabetes

Characteristics	Hazard ratio	95% Confidence interval	*P* ^‡^
Age (years)	1.02	1.01, 1.03	< 0.001
Sex			
Female	1.00	—	
Male	0.88	0.73, 1.06	0.181
Smoking	0.67	0.44, 1.02	0.064
Alcohol drinking	0.57	0.33, 0.97	0.037
Duration of type 2 diabetes (years)	0.99	0.97, 1.01	0.484
Body mass index (kg/m^2^)	0.96	0.92, 1.01	0.098
HbA1c level (%)	1.02	0.95, 1.10	0.553
Fasting blood glucose (mmol/L)	0.98	0.95, 1.01	0.165
Systolic blood pressure (mm Hg)	1.01	1.00, 1.01	0.151
Diastolic blood pressure (mm Hg)	1.01	0.99, 1.02	0.273
Low-density lipoprotein (mmol/L)	0.88	0.70, 1.10	0.256
High-density lipoprotein (mmol/L)	0.66	0.34, 1.29	0.222
Apolipoprotein A (g/L)	0.56	0.25, 1.24	0.153
Apolipoprotein B (g/L)	0.48	0.21, 1.10	0.083
Apolipoprotein E (mg/L)	0.99	0.98, 1.00	0.116
Lipoprotein(a)^**†**^ (mg/L)	0.9995	0.9982, 1.0009	0.482
Serum urea (mmol/L)	1.04	0.99, 1.09	0.155
Serum creatinine^**†**^ (µmol/L)	0.9992	0.9952, 1.0032	0.689
Serum uric acid^**†**^ (µmol/L)	0.9975	0.9963, 0.9987	< 0.001
Cystatin C (mg/L)	1.12	0.84, 1.49	0.426
Total protein^**†**^ (g/L)	0.9966	0.9818, 1.0116	0.653
Albumin (g/L)	0.99	0.97, 1.00	0.134
Total cholesterol (mmol/L)	0.96	0.88, 1.05	0.416
Triglyceride (mmol/L)	0.82	0.69, 0.98	0.030
Total bilirubin^**†**^ (µmol/L)	0.9980	0.9932, 1.0029	0.424
Direct bilirubin^**†**^ (µmol/L)	0.9985	0.9927, 1.0043	0.610
AST^**†**^ (U/L)	0.9999	0.9988, 1.0011	0.883
ALT^**†**^ (U/L)	0.9995	0.9980, 1.0010	0.500
AST/ALT	1.20	1.05, 1.36	0.007
Total bile acid^**†**^ (µmol/L)	0.9988	0.9946, 1.0030	0.573
Estimated glomerular filtration rate (CKD-EPI)^**†**^, mL/min	0.9968	0.9891, 1.0046	0.424
Glycated albumin (%)	1.01	1.00, 1.02	0.003
Platelet^**†**^ (10^9^)	0.9997	0.9984, 1.0009	0.602
White blood cell^**†**^ (10^9^)	0.9979	0.9651, 1.0317	0.900
Lymphocyte (10^9^)	0.80	0.69, 0.93	0.004
Neutrophil (10^9^)	1.01	0.98, 1.04	0.579
Erythrocyte (10^12^)	0.90	0.78, 1.04	0.142
Mean corpuscular hemoglobin concentration^**†**^ (g/L)	0.9951	0.9878, 1.0026	0.201

*Abbreviations:AST* Aspartate aminotransferase, *ALT* Alanine transaminase, *HbAlc* Haemoglobin A1c

^**†**^All hazard ratios equaling to 1.00 are reported with four decimal places to enhance precision and resolve rounding-related ambiguities in marginal associations

^‡^Only associations with *P* < 0.0013 are considered robust; Other nominally significant findings (*P* < 0.05) require independent validation

All variables included in the multivariable Cox regression models demonstrated statistical independence, with VIFs < 2 (range: 1.02–1.13; Supplementary Table 3), well below the conservative threshold of 5. While multivariable analysis identified elevated AST/ALT ratio (HR: 1.84, 95% CI: 1.16–2.92, *p* = 0.009) as an independent risk factor, the precision of the estimate warrants careful interpretation. As shown in the forest plot (Fig. 2), serum uric acid exhibited marginal significance (HR: 0.9970, 95% CI: 0.9942–0.9998, *p* = 0.037), suggesting limited clinical relevance despite statistical significance.

### Types of cancer among T2DM patients

**Fig. 3:**
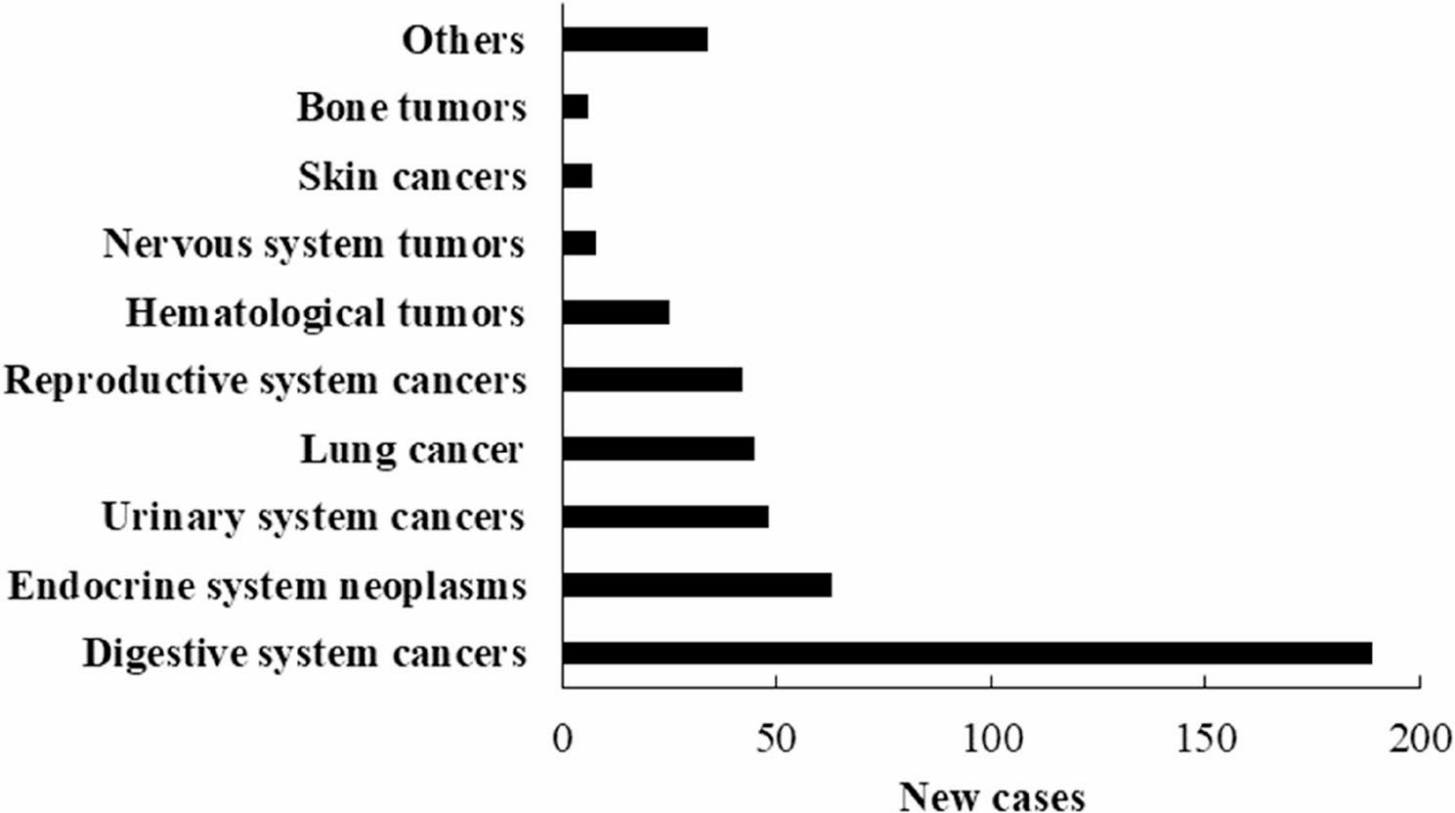
New cases of cancers among included patients with type 2 diabetes

Of the 3284 included patients, 467 (14.2%) were diagnosed with cancer during a median follow-up period of 27 months. According to Fig. 3, the most common type was digestive system cancers (189 cases, 5.8%), followed by endocrine system neoplasms (63, 1.9%), urinary system cancers (48, 1.5%), lung cancer (45, 1.4%), reproductive system cancers (42, 1.3%), hematological tumors (25, 0.8%), nervous system tumors (8, 0.2%), skin cancers (7, 0.2%), bone tumors (6, 0.2%) and other types of cancer.

### Risk factors for different types of cancer

To identify cancer type-specific risk factors, we conducted univariate and multivariate Cox proportional hazards analyses on cancers with > 1% incidence in the T2DM cohort. Given the limited sample size in certain subgroups, these analyses should be interpreted as exploratory. Despite this, our findings indicate distinct risk patterns for different types of cancer based on patient characteristics. Older patients were at a higher risk for digestive system (HR: 1.03, 95% CI: 1.01–1.04, *p* < 0.001), and urinary system cancers (HR: 1.04, 95% CI: 1.02–1.07, *p* < 0.001). A higher BMI was associated with a reduced risk of digestive system cancers (HR: 0.86, 95% CI: 0.79–0.94, *p* < 0.001). Serum urea showed a notable link to urinary system neoplasms (HR: 1.16, 95% CI: 1.08–1.26, *p* < 0.001). We also found that lower levels of serum uric acid and albumin, as well as higher levels of AST/ALT ratio were associated with digestive system cancers. Furthermore, elevated levels of glycated albumin was associated with endocrine system neoplasms (Table 3).

**Table 3:** Risk analysis of different cancer types using univariate Cox proportional hazards model in type 2 diabetes

Characteristics	Digestive system cancers		Lung cancer		Endocrine system neoplasms		Reproductive system cancers		Urinary system cancers	
	HR (95% CI)	*P* ^‡^	HR (95% CI)	*P* ^‡^	HR (95% CI)	*P* ^‡^	HR (95% CI)	*P* ^‡^	HR (95% CI)	*P* ^‡^
Age (years)	1.03 (1.01, 1.04)	< 0.001	1.04 (1.01, 1.07)	0.003	0.97 (0.96, 0.99)	0.005	0.99 (0.96, 1.01)	0.290	1.04 (1.02, 1.07)	< 0.001
Sex										
Female	—		—		—		—		—	
Male	1.22 (0.90, 1.65)	0.208	0.88 (0.48, 1.61)	0.670	0.89 (0.54, 1.47)	0.644	0 (0.00, Inf)	0.996	1.84 (0.95, 3.54)	0.068
Smoking	1.08 (0.64, 1.82)	0.772	0 (0.00, Inf)	0.995	0.51 (0.12, 2.11)	0.353	0 (0.00, Inf)	0.996	1.05 (0.37, 2.95)	0.930
Alcohol drinking	0.91 (0.48, 1.73)	0.768	0 (0.00, Inf)	0.996	0.35 (0.05, 2.56)	0.301	0 (0.00, Inf)	0.996	0.34 (0.05, 2.50)	0.289
Duration of type 2 diabetes (years)	0.98 (0.95, 1.01)	0.189	0.96 (0.90, 1.04)	0.310	0.93 (0.86, 1.01)	0.089	0.9 (0.81, 1.00)	0.060	1.06 (1.02, 1.11)	0.002
Body mass index (kg/m^2^)	0.86 (0.79, 0.94)	< 0.001	0.86 (0.72, 1.03)	0.096	0.99 (0.90, 1.10)	0.885	0.9 (0.76, 1.07)	0.221	1.04 (0.94, 1.14)	0.445
HbA1c level (%)	1.01 (0.89, 1.13)	0.899	1.24 (0.98, 1.58)	0.077	0.88 (0.71, 1.09)	0.229	1.14 (0.94, 1.38)	0.184	0.9951 (0.7769, 1.2747) ^†^	0.969
Fasting blood glucose (mmol/L)	0.99 (0.95, 1.04)	0.658	0.97 (0.88, 1.08)	0.609	0.91 (0.82, 1.00)	0.050	1.02 (0.95, 1.09)	0.592	0.95 (0.87, 1.05)	0.340
Systolic blood pressure (mm Hg)	0.9993 (0.9872, 1.0115) ^†^	0.907	1.0039 (0.9827, 1.0255) ^†^	0.721	1.02 (1.00, 1.04)	0.026	0.9953 (0.9733, 1.0179) ^†^	0.682	1.01 (0.99, 1.03)	0.438
Diastolic blood pressure (mm Hg)	1.0030 (0.9831, 1.0233) ^†^	0.772	1.0047 (0.9691, 1.0416) ^†^	0.799	1.04 (1.01, 1.07)	0.008	0.99 (0.95, 1.02)	0.483	1.01 (0.98, 1.05)	0.450
Low-density lipoprotein (mmol/L)	0.79 (0.53, 1.19)	0.263	1.16 (0.62, 2.19)	0.641	0.86 (0.55, 1.34)	0.495	1.08 (0.55, 2.12)	0.819	1.28 (0.63, 2.62)	0.497
High-density lipoprotein (mmol/L)	0.64 (0.20, 1.98)	0.437	1.17 (0.61, 2.23)	0.637	0.44 (0.11, 1.76)	0.246	1.23 (0.71, 2.13)	0.462	0.58 (0.05, 6.98)	0.665
Apolipoprotein A (g/L)	0.18 (0.05, 0.71)	0.014	0.97 (0.08, 11.3)	0.982	0.68 (0.14, 3.23)	0.628	16.3 (1.35, 197)	0.028	1.79 (0.10, 31.5)	0.690
Apolipoprotein B (g/L)	0.28 (0.06, 1.24)	0.094	0.66 (0.06, 7.55)	0.735	0.47 (0.09, 2.37)	0.358	0.86 (0.07, 11.2)	0.909	2.39 (0.19, 29.5)	0.498
Apolipoprotein E (mg/L)	0.9969 (0.9809, 1.0133) ^†^	0.711	0.95 (0.90, 1.01)	0.088	0.97 (0.94, 1.00)	0.041	1.02 (1.00, 1.03)	0.048	1.0007 (0.9717, 1.0312) ^†^	0.966
Lipoprotein(a) (mg/L)	0.9975 (0.9940, 1.0010) ^†^	0.158	1.0001 (0.9968, 1.0034) ^†^	0.960	1.0002 (0.9983, 1.0022) ^†^	0.832	0.9997 (0.9958, 1.0036) ^†^	0.884	0.9950 (0.9860, 1.0041) ^†^	0.283
Serum urea (mmol/L)	0.94 (0.84, 1.04)	0.205	0.99 (0.82, 1.20)	0.910	1.07 (0.96, 1.20)	0.195	1.01 (0.87, 1.18)	0.873	1.16 (1.08, 1.26)	< 0.001
Serum creatinine (µmol/L)	0.99 (0.98, 1.00)	0.026	0.9962 (0.9774, 1.0153) ^†^	0.692	1.0030 (0.9990, 1.0070) ^†^	0.144	0.98 (0.96, 1.00)	0.057	1.0038 (1.0006, 1.0070) ^†^	0.019
Serum uric acid (µmol/L)	0.99 (0.99, 1.00)	< 0.001	0.9955 (0.9911, 0.9999) ^†^	0.043	0.98 (0.96, 1.01)	0.255	0.9981 (0.9945, 1.0018) ^†^	0.310	1.0010 (0.9978, 1.0042) ^†^	0.535
Cystatin C (mg/L)	1.09 (0.66, 1.78)	0.747	0.65 (0.18, 2.38)	0.520	1.08 (0.50, 2.35)	0.841	0.84 (0.30, 2.31)	0.729	1.74 (1.18, 2.57)	0.005
Total protein (g/L)	0.97 (0.95, 0.99)	0.015	0.99 (0.95, 1.04)	0.760	1.01 (0.97, 1.05)	0.659	1.01 (0.97, 1.05)	0.695	1.05 (1.00, 1.09)	0.038
Albumin (g/L)	0.94 (0.91, 0.97)	< 0.001	1.0033 (0.9419, 1.0687) ^†^	0.919	1.08 (1.03, 1.14)	0.002	0.96 (0.91, 1.01)	0.141	1.01 (0.96, 1.07)	0.695
Total cholesterol (mmol/L)	0.93 (0.79, 1.09)	0.348	0.98 (0.74, 1.29)	0.879	0.94 (0.84, 1.06)	0.324	1.03 (0.85, 1.26)	0.733	0.95 (0.73, 1.24)	0.723
Triglyceride (mmol/L)	0.72 (0.51, 1.03)	0.072	0.47 (0.18, 1.19)	0.111	0.93 (0.71, 1.20)	0.561	0.97 (0.68, 1.39)	0.884	0.92 (0.56, 1.50)	0.732
Total bilirubin (µmol/L)	1.01 (1.00, 1.01)	0.002	0.97 (0.92, 1.02)	0.189	0.98 (0.95, 1.01)	0.161	0.95 (0.91, 1.00)	0.060	0.96 (0.91, 1.01)	0.081
Direct bilirubin (µmol/L)	1.01 (1.00, 1.01)	0.003	0.95 (0.87, 1.04)	0.315	0.92 (0.83, 1.01)	0.085	0.9 (0.79, 1.02)	0.089	0.91 (0.81, 1.02)	0.110
AST (U/L)	1.0010 (1.0001, 1.0019) ^†^	0.035	0.98 (0.96, 1.01)	0.157	0.99 (0.98, 1.01)	0.240	0.98 (0.95, 1.01)	0.118	0.98 (0.95, 1.01)	0.114
ALT (U/L)	1.0010 (1.0000, 1.0020) ^†^	0.044	0.99 (0.98, 1.01)	0.345	0.99 (0.99, 1.00)	0.249	0.99 (0.97, 1.00)	0.151	0.99 (0.97, 1.00)	0.126
AST/ALT	1.34 (1.17, 1.55)	< 0.001	0.67 (0.28, 1.63)	0.382	0.86 (0.47, 1.59)	0.628	1.2 (0.91, 1.57)	0.198	1.01 (0.62, 1.65)	0.968
Total bile acid (µmol/L)	1.01 (1.00, 1.01)	0.004	0.93 (0.85, 1.02)	0.125	0.98 (0.96, 1.01)	0.255	0.99 (0.97, 1.01)	0.373	0.97 (0.93, 1.02)	0.235
Estimated glomerular filtration rate (CKD-EPI), mL/min	0.9984 (0.9839, 1.0131) ^†^	0.831	0.99 (0.97, 1.02)	0.690	0.9993 (0.9801, 1.0189) ^†^	0.943	1.01 (0.99, 1.03)	0.207	0.98 (0.96, 0.99)	0.003
Glycated albumin (%)	1.01 (0.99, 1.03)	0.200	0.99 (0.92, 1.06)	0.707	1.01 (1.01, 1.02)	< 0.001	0.98 (0.91, 1.05)	0.485	1.01 (0.99, 1.03)	0.219
Platelet (10^9^)	0.9964 (0.9941, 0.9987) ^†^	0.002	1.0015 (0.9978, 1.0053) ^†^	0.423	1.0015 (0.9985, 1.0046) ^†^	0.317	1.0038 (1.0012, 1.0063) ^†^	0.004	1.0003 (0.9967, 1.0040) ^†^	0.860
white blood cell (10^9^)	0.98 (0.92, 1.04)	0.485	1.05 (0.96, 1.15)	0.266	0.95 (0.85, 1.05)	0.308	0.91 (0.80, 1.03)	0.128	1.06 (0.99, 1.14)	0.106
Lymphocyte (10^9^)	0.67 (0.51, 0.86)	0.002	0.82 (0.50, 1.35)	0.445	1.05 (0.72, 1.53)	0.817	0.68 (0.42, 1.09)	0.110	1.11 (0.74, 1.66)	0.613
Neutrophil (10^9^)	1.0046 (0.9482, 1.0645) ^†^	0.876	1.06 (0.97, 1.16)	0.197	0.94 (0.84, 1.06)	0.324	0.93 (0.81, 1.07)	0.305	1.06 (0.98, 1.14)	0.177
Erythrocyte (10^12^)	0.77 (0.62, 0.95)	0.016	0.81 (0.52, 1.28)	0.371	1.64 (1.05, 2.57)	0.030	0.75 (0.50, 1.11)	0.148	1.04 (0.67, 1.60)	0.869
Mean corpuscular hemoglobin concentration (g/L)	0.9981 (0.9859, 1.0105) ^†^	0.761	0.99 (0.96, 1.01)	0.305	1.01 (0.99, 1.03)	0.433	0.97 (0.96, 0.99)	0.008	1.01 (0.98, 1.03)	0.632

*Abbreviations:AST* Aspartate aminotransferase, *ALT* Alanine transaminase, *CI* Confidence interval, *HbAlc* Haemoglobin A1c, *HR* Hazard ratio

^**†**^All hazard ratios equaling to 1.00 are reported with four decimal places to enhance precision and resolve rounding-related ambiguities in marginal associations

^‡^Only associations with *P* < 0.0013 are considered robust; Other nominally significant findings (*P* < 0.05) require independent validation

To refine our findings, we further performed a multivariate analysis incorporating variables with *P* < 0.05. Apolipoprotein A levels exhibited a strong inverse association with digestive system cancers (HR: 0.01, 95% CI: 0.00–0.13, *p* < 0.001). Older age maintained a positive correlation with lung cancer (HR: 1.05, 95% CI: 1.01–1.08, *p* = 0.010). Elevated serum urea levels remained associated with urinary systemic cancers (HR: 1.21, 95% CI: 1.10–1.33, *p* < 0.001; Supplementary Table 4).

## Discussion

The association between T2DM and cancer risk is increasingly recognized. Antidiabetic medications, notably metformin and insulin, have been shown to independently influence cancer risk. Metformin, a first-line T2DM treatment, reduces the risks of liver and bile duct cancers [25], while insulin use is associated with a lower pancreatic cancer risk [26]. These observations suggest that T2DM may act as an independent cancer risk factor. Our retrospective cohort analysis revealed a notable 14.2% cancer incidence over a median follow-up period of 27 months, underscoring the urgent need for better cancer screening in this high-risk population. T2DM patients experienced an unchanged or even increased incidence of cancer over time [27]. Long-term hyperglycemia, insulin resistance, chronic inflammation, and dysregulated metabolic pathways appear central to this connection. Frequent hyperglycemia fuel cancer growth while triggering inflammation and oxidative stress [28]. Additionally, compensatory hyperinsulinemia may activate the insulin/IGF axis, stimulating cancer-promoting signaling pathways such as PI3K/Akt/mTOR [29–33]. Shared lifestyle factors such as obesity and unhealthy diets further link these diseases.

Older T2DM patients showed higher cancer risk, consistent with findings in colorectal, liver and pancreatic cancers [34–37]. Aging-related biological vulnerability and persistent metabolic disturbances in T2DM may rise cancer risk. Our male-dominated cohort (59%) could reflect gender disparities in cancer risk, potentially attributable to hormonal and genetic variations [38, 39].

Our study identified lower serum uric acid levels and elevated AST/ALT ratios as significant cancer risk factors in T2DM patients. Serum uric acid, an antioxidant end product of purine metabolism, presented conflicting evidence as a cancer protector in T2DM. Elevated uric acid was linked to cancer risk via inflammation [20, 40], while low levels may also increase risk through its antioxidant and immunomodulatory roles [41, 42], suggesting a U-shaped relationship with both extremes elevating cancer risk [43]. The higher AST/ALT ratio, indicating liver stress, has been identified as a cancer risk factor, consistent with findings from a prospective study of the general population [44]. This association is likely attributed to diabetes-related metabolic dysfunction fostering a tumorigenic microenvironment through insulin resistance and chronic inflammation [19]. However, these mechanistic speculations are hypothesis-generating at this stage, as our study design cannot establish causality. Future preclinical models (e.g., organoid or animal studies) are needed to test whether liver - specific metabolic stress directly promotes carcinogenesis.

Digestive system cancers were the most common in our cohort (5.8% of cases), aligning with studies showing higher gastrointestinal cancer rates in diabetes [45]. This convergence may originate from shared drivers like obesity, chronic inflammation, and insulin resistance [46]. In terms of cancer type distribution, age significantly influenced risk profiles. Older patients exhibited a higher risk for digestive, lung, and urinary system cancers, whereas younger individuals appeared more susceptible to endocrine system neoplasms. Such age-related differences imply that distinct biological mechanisms may drive cancer development in younger versus older individuals with T2DM, emphasizing the need for personalized screening strategies in diabetes care.

Our study also revealed some other connections between diabetes-related factors and specific cancer risk. For example, longer diabetes duration was tied to uninary system cancers, suggesting chronic hyperglycemia on oncogenic processes. Surprisingly, lower BMI increased digestive system cancers, implying that metabolic dysregulation, rather than obesity alone, drives carcinogenic process. These associations warrant further investigation to enhance earlier detection and intervention strategies.

Interestingly, apolipoprotein A, a key component of high density lipoprotein, showed contrasting effects across cancer types: it reduced digestive system cancer risk but increased reproductive cancer risk, highlighting tissue-specific effects [47]. Even after adjusting for covariates in multivariate models, lower apolipoprotein A levels remained associated with digestive system cancers. Apolipoprotein A might promote apoptosis and inhibit hepatocellular carcinoma cell proliferation by downregulating the MAPK pathway [48]. Though the expression of apolipoprotein A varied across cancer types [47], it has been shown to suppress colon cancer [49] and hepatocellular carcinoma [48], consistent with our observed protective effects in T2DM patients. Meanwhile, higher serum urea levels tied to urinary cancers but paradoxically linked to lower overall cancer risk [50], indicating the complex interplay between diabetes-related kidney changes and cancer development. However, subgroup analyses for cancers with limited sample sizes (e.g., lung cancer: 45 cases) should be viewed as hypothesis-generating due to limited statistical power, and wide CIs in some associations (e.g., apolipoprotein A for reproductive cancers: HR 16.3, 95% CI 1.35–197) underscore substantial uncertainty in these estimates. These preliminary associations require validation in larger, independent cohorts.

These findings advocate for integrating cancer risk assessment into routine diabetes care. Clinicians should prioritize regular screenings for high-risk T2DM patients—particularly older individuals or those with low uric acid levels and abnormal liver enzyme ratios. Lifestyle interventions targeting weight, alcohol, and metabolic health remain crucial, while expanding metabolic monitoring to include uric acid and liver function tests could refine risk prediction. For example, abnormal liver enzyme ratios could guide liver cancer surveillance. These practical strategies highlight how simple blood tests already used in diabetes management might double as cancer early-warning tools. While prior studies have established associations between specific biomarkers and cancer risk in T2DM [20–22] or developed machine learning models for cancer risk prediction [5], our multivariate model provides an interpretable model by integrating accessible, low-cost biomarkers, enabling clinically feasible cancer risk stratification in patients with T2DM.

While our study provides valuable insights, it has several limitations. Its observational design precludes causal inference, and unmeasured lifestyle factors like physical activity and diet may introduce residual confounding. The single-center design, coupled with the absence of both an independent validation cohort and external replication, may limit the generalizability. Potential overfitting due to limited cancer events relative to covariates necessitates cautious interpretation until replication in larger, multiethnic cohorts. And relying on electronic medical records risks diagnostic misclassification. Excluding patients with advanced comorbidities, despite improving internal validity, may introduce selection bias and underestimate cancer risk in multimorbid populations. However, sensitivity analyses retaining patients with severe respiratory diseases and hypertension showed consistent biomarker-cancer associations, suggesting clinically relevant across disease stages. Additionally, the relatively short follow-up period may underestimate long-term cancer risks, necessitating future studies with extended follow-up. Moreover, the marginal significance of uric acid and wide CIs for some HRs (e.g., male sex, apolipoprotein A) require validation in other cohorts.

## Conclusion

Our retrospective cohort study observed associations between abnormal liver function ratios and specific lipid biomarkers with cancer risk among patients with T2DM. While these findings indicate the potential utility of metabolic profiling for risk stratification, their causal relevance remain unclear, and their clinical translation requires validation in prospective designed, multi-ethnic cohorts with extended follow-up periods.

## Supplementary Information

Supplementary Material 1.

## Data Availability

The datasets analyzed are available from the corresponding author on reasonable request. The details of variables are provided as Supplementary Table 5.

## Abbreviations

ALT: Alanine transaminase
AST: Aspartate aminotransferase
BMI: Body mass index
CIs: Confidence intervals
HbAlc: Glycated haemoglobin A1c
HRs: Hazard ratios
IQR: Interquartile range
T2DM: Type 2 diabetes mellitus
