# Supplementary material for: Risk factors for cancer among patients with type 2 diabetes: a retrospective cohort study
Source: BMC Cancer. 2025 Jul 1;25:1059. doi: 10.1186/s12885-025-14483-4 (PMC12211339; doi:10.1186/s12885-025-14483-4)
Supplement: Supplementary file 1 — Supplementary Material 1. [file 12885_2025_14483_MOESM1_ESM.docx]

**Supplementary Table 1. The result of Proportional Hazards Assumption Test using Schoenfeld Residuals.**

| **Variables** | ***P*** |
| --- | --- |
| Age | 0.92 |
| Sex | 0.26 |
| Smoking | 0.67 |
| Alcohol drinking | 0.67 |
| Duration of type 2 diabetes | 0.52 |
| Body mass index | 0.11 |
| HbA1c level | 0.40 |
| Fasting blood glucose | 0.09 |
| Systolic blood pressure | 0.17 |
| Diastolic blood pressure | 0.75 |
| Low-density lipoprotein | 0.97 |
| High-density lipoprotein | 0.38 |
| Apolipoprotein A | 0.57 |
| Apolipoprotein B | 0.91 |
| Apolipoprotein E | 0.24 |
| Lipoprotein(a) | 0.36 |
| Serum urea | 0.27 |
| Serum creatinine | 0.21 |
| Serum uric acid | 0.09 |
| Cystatin C | 0.37 |
| Total protein | 0.40 |
| Albumin | 0.96 |
| Total cholesterol | 0.73 |
| Triglyceride | 0.69 |
| Total bilirubin | 0.88 |
| Direct bilirubin | 0.57 |
| AST | 0.44 |
| ALT | 0.51 |
| AST/ALT | 0.24 |
| Total bile acid | 0.24 |
| Estimated glomerular filtration rate (CKD-EPI) | 0.19 |
| Glycated albumin | 0.32 |
| Platelet | 0.12 |
| White blood cell | 0.42 |
| Lymphocyte | 0.69 |
| Neutrophil | 0.68 |
| Erythrocyte | 0.54 |
| Mean corpuscular hemoglobin concentration | 0.40 |
| Global | 0.73 |

Abbreviations: AST, aspartate aminotransferase; ALT, alanine transaminase.

**Supplementary Table 2. The Cox model analysis for cancer risk in patients retaining those with severe respiratory disease or hypertension.**

| **Characteristics** | **Hazard ratio** | **95% Confidence interval** | ***P*** |
| --- | --- | --- | --- |
| Age (years) | 1.02 | 1.01, 1.03 | **<0.001** |
| Sex |  |  |  |
| Female | 1.00 | — |  |
| Male | 0.89 | 0.74, 1.07 | 0.222 |
| Smoking | 0.68 | 0.46, 1.03 | 0.069 |
| Alcohol drinking | 0.60 | 0.36, 0.99 | **0.045** |
| Duration of type 2 diabetes (years) | 0.99 | 0.97, 1.01 | 0.373 |
| Body mass index (kg/m^2^) | 0.96 | 0.91, 1.01 | 0.089 |
| HbA1c level (%) | 1.01 | 0.94, 1.09 | 0.777 |
| Fasting blood glucose (mmol/L) | 0.98 | 0.95, 1.00 | 0.098 |
| Systolic blood pressure (mm Hg) | 1.01 | 1.00, 1.01 | 0.070 |
| Diastolic blood pressure (mm Hg) | 1.01 | 1.00, 1.02 | 0.143 |
| Low-density lipoprotein (mmol/L) | 0.89 | 0.71 1.11 | 0.302 |
| High-density lipoprotein (mmol/L) | 0.63 | 0.32 1.22 | 0.170 |
| Apolipoprotein A (g/L) | 0.59 | 0.26 1.30 | 0.187 |
| Apolipoprotein B (g/L) | 0.51 | 0.22 1.15 | 0.102 |
| Apolipoprotein E (mg/L) | 0.99 | 0.98, 1.00 | 0.108 |
| Lipoprotein(a) (mg/L) | 1.00 | 1.00, 1.00 | 0.508 |
| Serum urea (mmol/L) | 1.04 | 0.99, 1.09 | 0.12 |
| Serum creatinine (μmol/L) | 1.00 | 1.00, 1.00 | 0.788 |
| Serum uric acid (μmol/L) | 1.00 | 1.00, 1.00 | **<0.001** |
| Cystatin C (mg/L) | 1.11 | 0.86 1.43 | 0.435 |
| Total protein (g/L) | 1.00 | 0.98, 1.01 | 0.938 |
| Albumin (g/L) | 0.99 | 0.97, 1.01 | 0.245 |
| Total cholesterol (mmol/L) | 0.96 | 0.88, 1.05 | 0.365 |
| Triglyceride (mmol/L) | 0.83 | 0.70, 0.98 | **0.031** |
| Total bilirubin (μmol/L) | 1.00 | 0.99, 1.00 | 0.659 |
| Direct bilirubin (μmol/L) | 1.00 | 0.99, 1.00 | 0.886 |
| AST (U/L) | 1.00 | 1.00, 1.00 | 0.997 |
| ALT (U/L) | 1.00 | 1.00, 1.00 | 0.635 |
| AST/ALT | 1.17 | 1.02, 1.34 | **0.021** |
| Total bile acid (μmol/L) | 1.00 | 1.00, 1.00 | 0.868 |
| Estimated glomerular filtration rate (CKD-EPI), mL/min | 1.00 | 0.99, 1.00 | 0.436 |
| Glycated albumin (%) | 1.01 | 1.00, 1.02 | **0.005** |
| Platelet (10^9^) | 1.00 | 1.00, 1.00 | 0.789 |
| White blood cell (10^9^) | 1.00 | 0.97, 1.03 | 0.900 |
| Lymphocyte (10^9^) | 0.80 | 0.69, 0.93 | **0.004** |
| Neutrophil (10^9^) | 1.01 | 0.98, 1.04 | 0.537 |
| Erythrocyte (10^12^) | 0.91 | 0.79, 1.04 | 0.177 |
| Mean corpuscular hemoglobin concentration (g/L) | 1.00 | 0.99, 1.00 | 0.186 |

Abbreviations: AST, aspartate aminotransferase; ALT, alanine transaminase; HbAlc, haemoglobin A1c.

**Supplementary Table 3. Variance inflation factors (VIFs) of variables involved in the multivariable Cox regressions analysis for cancer risk factors in patients with type 2 diabetes.**

| **Variables** | **VIF** |
| --- | --- |
| Age | 1.077 |
| Alcohol drinking | 1.019 |
| Serum uric acid | 1.118 |
| Triglyceride | 1.108 |
| AST/ALT | 1.067 |
| Glycated albumin | 1.126 |
| Lymphocyte | 1.093 |

Abbreviations: AST, aspartate aminotransferase; ALT, alanine transaminase.

**Supplementary Table 4. Multivariate Cox’s proportional hazards model analysis for type-specific cancer risk in type 2 diabetes by backward regression.**

| **Characteristics** | **Digestive system cancers** | | **Lung cancer** | | **Urinary system cancers** | |
| --- | --- | --- | --- | --- | --- | --- |
|  | **HR (95% CI)** | ***P*** | **HR (95% CI)** | ***P*** | **HR (95% CI)** | ***P*** |
| Age (years) |  |  | 1.05 (1.01, 1.08) | 0.010 |  |  |
| Duration of type 2 diabetes (years) |  |  |  |  | 1.06 (1.01, 1.12) | 0.024 |
| Body mass index (kg/m^2^) | 0.78 (0.65, 0.92) | 0.004 |  |  |  |  |
| Apolipoprotein A (g/L) | 0.01 (0.00, 0.13) | <0.001 |  |  |  |  |
| Serum urea (mmol/L) |  |  |  |  | 1.21 (1.10, 1.33) | <0.001 |
| Serum uric acid (μmol/L) |  |  | 0.9954 (0.9909, 0.9999) ^†^ | 0.047 |  |  |
| Platelet (10^9^) | 0.99 (0.99, 1.00) | 0.025 |  |  |  |  |

^†^All hazard ratios equaling to 1.00 are reported with four decimal places to enhance precision and resolve rounding-related ambiguities in marginal associations.

Abbreviations: CI, confidence interval; HbAlc, haemoglobin A1c; HR, hazard ratio.

**Supplementary Table 5. Variables involved in this analysis.**

| **Variable name** | **Measurement unit** | **Data type** | **Normal reference ranges** | **Value ranges or categories** | **Categorical value definition** |
| --- | --- | --- | --- | --- | --- |
| Age | Year | Numerical | - | 14-93 | - |
| Sex | - | Categorical | - | 1, 2 | 1 (Male), 2 (Female) |
| Smoking | - | Categorical | - | 0, 1 | 0 (“No”, former /never-smokers), 1 (“Yes”, current smokers) |
| Alcohol drinking | - | Categorical | - | 0, 1 | 0 (“No”, never /former /occasional drinker), 1 (“Yes”, regular drinking) |
| Body mass index | kg/m^2^ | Numerical | 18.5 - 24.9 | 12.9-53.4 | - |
| Systolic blood pressure | mm Hg | Numerical | 90-139 | 63-200 | - |
| Diastolic blood pressure | mm Hg | Numerical | 60-89 | 30-118 | - |
| HbA1c level | % | Numerical | <7.0 | 3.0-16.1 | - |
| Fasting blood glucose | mmol/L | Numerical | 3.9-6.1 | 2.2-45.6 | - |
| Low-density lipoprotein | mmol/L | Numerical | 0-3.37 | 0.32-7.07 | - |
| High-density lipoprotein | mmol/L | Numerical | 1.16-1.42 | 0.18-11.13 | - |
| Apolipoprotein A | g/L | Numerical | 1.00-1.60 | 0.09-2.10 | - |
| Apolipoprotein B | g/L | Numerical | 0.60-1.10 | 0.21-2.16 | - |
| Apolipoprotein E | mg/L | Numerical | 30.5-48.5 | 9.2-314.3 | - |
| Lipoprotein(a) | mg/L | Numerical | 0-300 | 1.6-2470.0 | - |
| Duration of type 2 diabetes | Year | Numerical | - | 0-35 | - |
| Estimated glomerular filtration rate (CKD-EPI) | mL/min | Numerical | >90 | 6.79-225.43 | - |
| Serum urea | mmol/L | Numerical | 3.60-9.50 | 1.26-26.22 | - |
| Serum creatinine | μmol/L | Numerical | 57-111 | 19.0-792.6 | - |
| Serum uric acid | μmol/L | Numerical | 155-357 | 40.5-924.0 | - |
| Triglyceride | mmol/L | Numerical | <2.30 | 0.24-31.6 | - |
| Total cholesterol | mmol/L | Numerical | <5.17 | 1.04-30.72 | - |
| Platelet | 10^9^ | Numerical | 125-350 | 2-1026 | - |
| Albumin | g/L | Numerical | 40.0-55.0 | 17.8-64.6 | - |
| Total protein | g/L | Numerical | 65.0-85.0 | 35.1-100.8 | - |
| Cystatin C | mg/L | Numerical | 0.510-1.090 | 0.09-7.32 | - |
| Total bilirubin | μmol/L | Numerical | 3.4-17.1 | 0-558.40 | - |
| Direct bilirubin | μmol/L | Numerical | 0.0-3.4 | 0-461.7 | - |
| AST | U/L | Numerical | 15-40 | 5.4-1985.0 | - |
| ALT | U/L | Numerical | 9-50 | 1.0-2683.0 | - |
| AST/ALT | - | Numerical | - | 0.1-18.0 | - |
| Total bile acid | μmol/L | Numerical | 0.0-10.0 | 0.1-549.4 | - |
| Lymphocyte | 10^9^ | Numerical | 1.1-3.2 | 0.37-12.37 | - |
| Neutrophil | 10^9^ | Numerical | 1.8-6.3 | 0.21-33.71 | - |
| Mean corpuscular hemoglobin concentration | g/L | Numerical | 316-354 | 254-419 | - |
| White blood cell | 10^9^ | Numerical | 3.5-9.5 | 0.23-37.54 | - |
| Glycated albumin | % | Numerical | 11.0-16.0 | 4.40-344.65 | - |
| Erythrocyte | 10^12^ | Numerical | 4.3-5.8 | 0.67-7.09 | - |

**Supplementary Figure 1. Cumulative incidence by follow-up duration.**


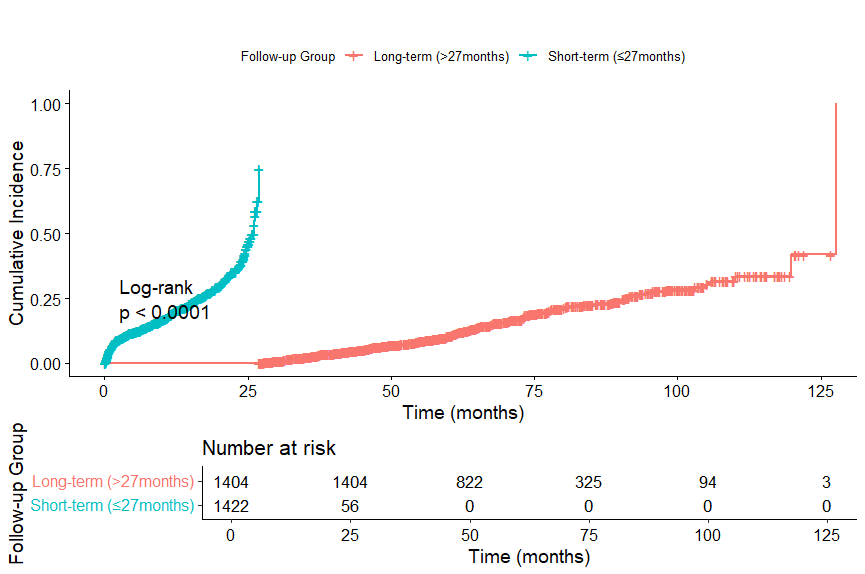


**Data Source and Quality Assurance**

All data were collected from the electronic medical records system. To ensure the data quality, we took several steps to minimize bias in our retrospective data collection. First, we used a predefined set of inclusion and exclusion criteria to select the study population. This helped to ensure that the sample was representative of the target population. Second, we used a standardized data extraction form to collect the relevant information from the electronic health records by trained research assistants. This reduced the potential for variability in data collection.

**Statistical Code Availability**

The R scripts used for multivariable Cox regression and forest plot generation are available upon reasonable request to the corresponding author.

**Supplementary Table 6. STROBE checklist.**

| **Item No.** | **STROBE Guideline** | **Location in Manuscript** |
| --- | --- | --- |
| **Title & Abstract** | | |
| 1 | (a) Indicate the study’s design with a commonly used term in the title or the abstract  (b) Provide in the abstract an informative and balanced summary of what was done and what was found | Title: “Risk factors for cancer among patients with type 2 diabetes: a retrospective cohort study”  Abstract: Section “Methods/Results/Conclusions” |
| **Introduction** | | |
| 2 | Explain the scientific background and rationale for the investigation being reported | Background: page 3-4 |
| 3 | State specific objectives, including any prespecified hypotheses | Background: Last paragraph (page 4) |
| **Methods** | | |
| 4 | Present key elements of study design early in the paper | Methods: “Participants” (Section 2.1) |
| 5 | Describe the setting, locations, and relevant dates, including periods of recruitment, exposure, follow-up, and data collection | Methods: “Participants” (Section 2.1), “Data collection” (Section 2.2) |
| 6 | (a) Give the eligibility criteria, and the sources and methods of selection of participants.  (b) For matched studies, give matching criteria and number of exposed and unexposed | Methods: “Participants” (Section 2.1)  Figure 1 |
| 7 | Clearly define all outcomes, exposures, predictors, potential confounders, and effect modifiers. Give diagnostic criteria, if applicable | Methods: “Data collection” (Section 2.2), “Follow up” (Section 2.3)  Supplementary Table 5 |
| 8 | For each variable of interest, give sources of data and details of methods of assessment (measurement). Describe comparability of assessment methods if there is more than one group | Methods: “Data collection” (Section 2.2) |
| 9 | Describe any efforts to address potential sources of bias | Methods: “Participants” (Section 2.1), “Follow up” (Section 2.3) |
| 10 | Explain how the study size was arrived at | Methods: “Participants” (Section 2.1) |
| 11 | Explain how quantitative variables were handled in the analyses. If applicable, describe which groupings were chosen and why | Methods: “Statistical analysis” (Section 2.4) |
| 12 | (a) Describe all statistical methods (b) Describe any methods used to examine subgroups and interactions (c) Explain missing data handling  (d) If applicable, explain how loss to follow-up was addressed (e) Describe sensitivity analyses | Methods: “Participants” (Section 2.1), “Statistical analysis” (Section 2.4) Supplementary Table 2 (Sensitivity Analysis) |
| **Results** | | |
| 13 | (a) Report numbers of individuals at each stage of study—eg numbers potentially eligible, examined for eligibility, confirmed eligible, included in the study, completing follow-up, and analysed  (b) Give reasons for non-participation at each stage  (c) Consider use of a flow diagram | Results: “Participants” (Section 3.1)  Figure 1 (flow diagram) |
| 14 | (a) Give characteristics of study participants (eg demographic, clinical, social) and information on exposures and potential confounders  (b) Indicate number of participants with missing data for each variable of interest  (c) Summarise follow-up time (eg, average and total amount) | Results: “Participants” (Section 3.1)  Table 1 (baseline characteristics) |
| 15 | Report numbers of outcome events or summary measures over time | Results: “Participants” (Section 3.1) |
| 16 | (a) Give unadjusted estimates and, if applicable, confounder-adjusted estimates and their precision (eg, 95% confidence interval). Make clear which confounders were adjusted for and why they were included  (b) Report category boundaries when continuous variables were categorized  (c) If relevant, consider translating estimates of relative risk into absolute risk for a meaningful time period | Results: “Risk factors for cancer among T2DM patients” (Section 3.2)  Table 2 (Univariate Cox’s proportional hazards model analysis) |
| 17 | Report other analyses done—eg analyses of subgroups and interactions, and sensitivity analyses | Results: “Risk factors for different types of cancer” (Section 3.4)  Table 3 (Risk analysis of different cancer types using univariate Cox proportional hazards model)  Supplementary Table 2 (Sensitivity Analysis) Supplementary Table 1 (PH Assumption) Supplementary Table 3 (Multicollinearity) |
| **Discussion** | | |
| 18 | Summarise key results with reference to study objectives | Discussion: First paragraph (page 14) |
| 19 | Discuss limitations of the study, taking into account sources of potential bias or imprecision. Discuss both direction and magnitude of any potential bias | Discussion: Last paragraph (page 14) |
| 20 | Give a cautious overall interpretation of results considering objectives, limitations, multiplicity of analyses, results from similar studies, and other relevant evidence | Conclusion (Section 5) |
| 21 | Discuss the generalisability (external validity) of the study results | Discussion: Last paragraph (page 14) |
| **Other Information** | | |
| 22 | Give the source of funding and the role of the funders for the present study and, if applicable, for the original study on which the present article is based | Funding (page 16) |
